# Solving the regulation puzzle of periderm development using advances in fruit skin

**DOI:** 10.3389/fpls.2022.1006153

**Published:** 2022-09-29

**Authors:** Yue-zhi Wang, Mei-song Dai, Dan-ying Cai, Ze-bin Shi

**Affiliations:** Institute of Horticulture, Zhejiang Academy of Agricultural Sciences, Hangzhou, China

**Keywords:** periderm, secondary development, abiotic stress, programmed cell death, fruit skin

## Abstract

Periderm protects enlarged organs of most dicots and gymnosperms as a barrier to water loss and disease invasion during their secondary growth. Its development undergoes a complex process with genetically controlled and environmental stress-induced characters. Different development of periderm makes the full and partial russet of fruit skin, which diverges in inheritance with qualitative and quantitative characters, respectively, in pear pome. In addition to its specific genetics, fruit periderm has similar development and structure as that of stem and other organs, making it an appropriate material for periderm research. Recently, progress in histochemical as well as transcriptome and proteome analyses, and quantitative trait locus (QTL) mapping have revealed the regulatory molecular mechanism in the periderm based on the identification of switch genes. In this review, we concentrate on the periderm development, propose the conservation of periderm regulation between fruit and other plant organs based on their morphological and molecular characteristics, and summarize a regulatory network with the elicitors and repressors for the tissue development. Spontaneous programmed-cell death (PCD) or environmental stress produces the original signal that triggers the development of periderm. Spatio-temporal specific PCD produced by *PyPPCD1* gene and its homologs can play a key role in the coordinated regulation of cell death related tissue development.

## Introduction

Periderm replaces the cuticle to protect growing organs of most dicots and gymnosperms during their secondary growth (Wunderling et al., 2018). The formation of periderm is a common phenomenon in stems and roots of dicotyledons and gymnosperms that increase in thickness by the secondary growth (Campilho et al., 2020). The cork cambium is re-differentiated in the vertical direction with outwardly to cork layer and inwardly to endodermis (Graça, 2015; Macnee et al., 2020). The formation of cork layer includes rapid cell enlargement and suberized cell walls, followed by cell death (Daneva et al., 2016). Periderm development is extremely complex and it differs among diverse species, genotypes and organs, and it is also characterized by developmental stages, seasonal changes, and environmental stresses (Lulai and Corsini, 1998; Caritat et al., 2000; Neubauer et al., 2012; Thangavel et al., 2016). Because of its importance for plant performance and survival, periderm has noticeably attracted attention, and the resulting studies have concentrated on the chemical composition of cork and high-resolution transcript profilings that have provided a reliable basis for periderm development (Vishwanath et al., 2015; Ragni and Greb, 2018; Campilho et al., 2020; Macnee et al., 2020).

Periderm development has been reported to be significantly associated with regulation of switch genes, which could be triggered during plant secondary growth. The identification of switch genes is the key to reveal the molecular mechanism for controlling the trait formation (Wang et al., 2016; Wang et al., 2022). However, obtaining genetic material has been difficult as the PCD mutations are often fatal (Wang et al., 2022). In addition to acting as the secondary protective tissue, periderm also affects the quality of fruits, such as that of pear, apple, and kiwi, in which the outmost cork layer in fruit skin affects the fruit appearance, storage capability, and shelf life. The genetic differentiation in the periderm development of fruit makes it highly appropriate for the study of biochemical profiles and molecular regulation of periderm (Wang et al., 2016; Macnee et al., 2020). Except for histochemical analysis, integrative transcriptome and proteome analysis and quantitative trait locus (QTL) mapping have been carried out for the russet trait made by periderm development in the fruit skin (Yamamoto et al., 2014; Falginella et al., 2015; Lashbrooke et al., 2015; Heng et al., 2016; Legay et al., 2016; Shi et al., 2019; Wang et al., 2020). In particular, the switch genes for fruit periderm development and the functional conservation of the homologs among different species (Lashbrooke et al., 2015; Wang et al., 2022) showed to have multiple important implications to reveal the regulatory mechanisms underlying periderm development. In the present review, we concentrate on the progresses of periderm development research in fruit skin, with an emphasis on exploring the original signal and the underlying regulatory mechanisms.

## Signals for fruit periderm development

Orchard factors that induce fruit periderm development to russet include the expansion-growth-induced strain and environmental stresses (Winkler et al., 2022). Partially russet development is stress inducible, and it can be repressed by rain sheltered cultivation and eliminated by fruit bagging (Wang et al., 2016; Shi et al., 2019; Zhang et al., 2021). Similar effect of bagging has been observed in other fruits (Winkler et al., 2022). These results suggest that expansion-growth-induced strain does not cause russeting independently in the absence of stress or certain environmental factors. Either fruit bagging or rain sheltered cultivation continuously provides an appropriate growing condition for fruits. However, when environmental changes exceed the allowable ranges that are required for the appropriate growth condition, stress is produced and periderm development is triggered. The dehydration caused by transpiration with inappropriate water supply can be the main environmental stress leading to the fruit russet formation (Athoo et al., 2020; Chen et al., 2020; Khanal et al., 2021).

**Figure 1:**
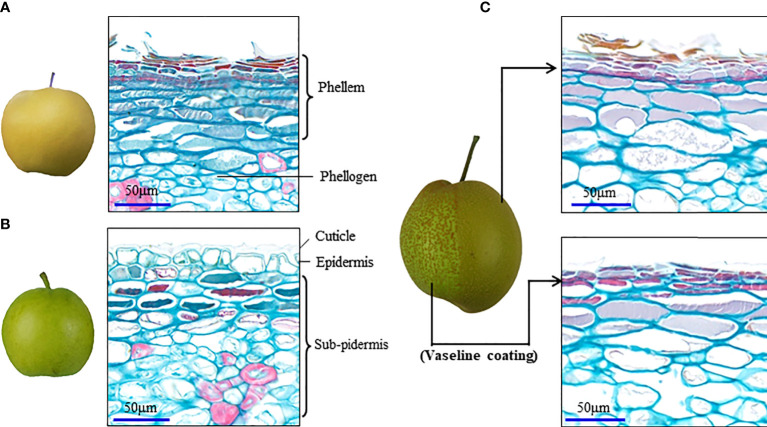
Vaseline coating repressed periderm development in the russet fruit skin of sand pear. **(A, B)** Naturally different periderm development between russet and green fruit skin of sand pear after 80 days of anthesis in normal growth conditions. **(C)** Vaseline coating on part of the fruit skin of sand pear that repressed the periderm development in the treatment position.

In addition to the type of induction by environmental stress, russeting is also formed by periderm development in fruit of some pear cultivars, which shows a dominant gene control and cannot be eliminated by fruit bagging (Wang et al., 2016). Histochemical staining at different development stages of pear fruit skin has demonstrated clearly spontaneous fruit periderm formation, including the steps of cell suberization and PCD (Macnee et al., 2020; Wang et al., 2022). For a long time, the regulatory relationship between the cell suberization and the PCD in the periderm development was not clearly documented until the switch gene for the russet formation was cloned in pear fruit skin (Wang et al., 2022). A study suggested that PCD in pear fruit skin has knock-on effects in terms of exocarp integrity with regards to gas-liquid exchange, resulting in the response of periderm development to the stress or environmental changes. High levels of oxygen and moisture play an important role in the fruit russet formation (Winkler et al., 2022). The H_2_O_2_ generated during polyamine metabolism in pear fruit skin was supposed to contribute to the fruit russet formation (Heng et al., 2016). The spontaneous periderm development can also be repressed when the fruit surface is coated with Vaseline before the fruit russet formation (**Figure 1**). A difference of Vaseline coating to fruit bagging treatment is that it can prevent the direct contact of fruit skin to oxygen. Thus, oxygen is considered as the necessary environmental factor for response of periderm development triggered by stress or a change in the environment.

Wounding induces changes in cytokinin and auxin content in potato tuber, followed by the wound periderm formation (Lulai et al., 2016). In the cellular de-differentiation analysis, auxin and cytokinin induce reentry into S-phase and proliferation of the mesophyll cells, and auxin may induce re-differentiation (Valente et al., 1998). Stress induces plant somatic embryogenesis to acquire some features of stem cells, which has been discussed widely as a fundamental theme in a recent issue of Biochimica et Biophysica Acta (the Special Issue: Stress as a fundamental theme in cellplasticity. 2015). The fruit susceptibility to russeting varies with developmental stages (Knoche et al., 2011; Khanal et al., 2021; Winkler et al., 2022). The de-differentiation potential of somatic cells to meristem depends on cell types and cell developmental stages, which can explain why periderm or russeting is more easily induced in the early developmental stage.

Except for initiating periderm development with a pathway similar to that of stress, signaling molecules triggering PCD or produced during the process of PCD may directly regulate the secondary metabolism for spontaneous peridermogenesis in adjacent cells (Wang et al., 2022). As a major component of biological membranes, lipids also serve as substrates for generating numerous signaling molecules involved in tolerance of and responses to biotic and abiotic stresses (Ali et al., 2022; Haslam and Feussner, 2022). Lipid biosynthesis, metabolism, and oxidation-reduction have been reported to be regulated, and the structure of plasma membrane has been altered and disrupted during the PCD of pear fruit skin (Wang et al., 2020; Wang et al., 2022).

## Genetic characteristics and gene mapping of fruit russet

**Figure 2:**
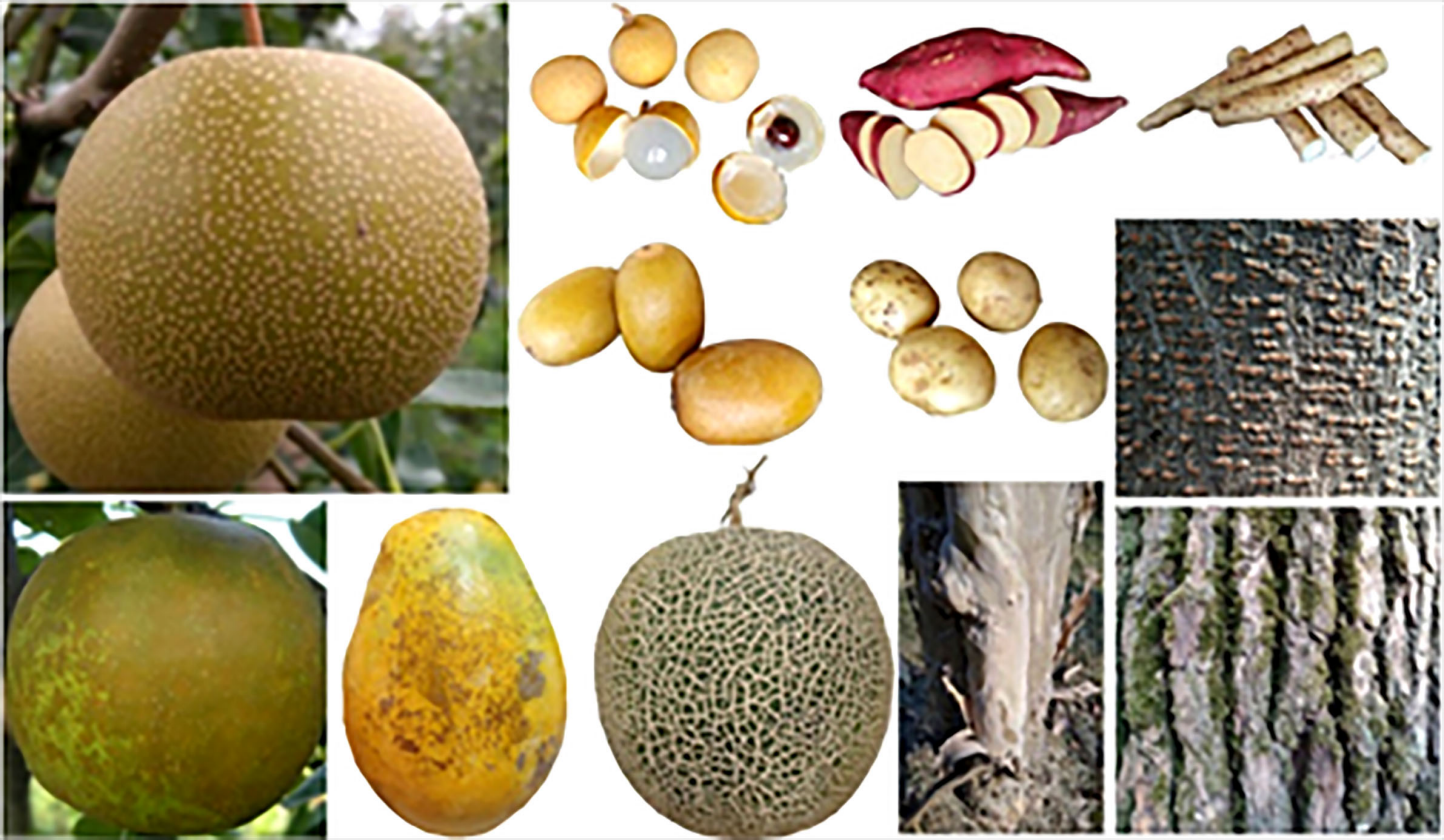
Appearance of different types of periderm.

As mentioned above, fruit russet made by periderm development is enriched in phenotypic variation. Such as that observed in pear, kiwifruit and apple, there exist both types of periderm that either completely or partially covers the fruit (Falginella et al., 2015; Lashbrooke et al., 2015; Wang et al., 2016; Macnee et al., 2020). The similar outmost periderm was also found in other storage organs as that of Potato (*Solanum tuberosum*), Sweet potato (*Ipomea batatas*), Cassava (*Manihot esculenta*), and Yam (*Rhizoma Dioscoreae*) (**Figure 2**). The inheritance of pear fruit russet has been studied for long. Early studies speculated two loci (*R* and *I*) controlling pear fruit skin russet, in which the *R* locus had the dominant effect on cork layer development, and the *I* locus had the dominant effect on cork layer suppression (Katayama et al., 2006; Song et al., 2010). Combined with normal distribution of the semi-russet in cross population and bi-directional russet mutations between smooth and russet exocarp, Wang et al. (2016) clustered pear peridermal fruits with qualitative characterization (full russet or complete peridermal skin) and quantitative characterization (semi-russet, partially peridermal skin). The two traits of full russet and semi-russet are inherited independently, while the former obscures the latter when they are co-inherited. A conservative dominant gene controlling the russet of pear fruit skin has been mapped at the end of the linkage group 8, which was also analyzed as a major QTL in several studies (Inoue et al., 2006; Song et al., 2010; Yamamoto et al., 2014; Kumar et al., 2017; Takeuchi et al., 2021; Wang et al., 2022). QTL mapping using different segregating progenies also led to the identification of one major determinant on the linkage group 12, and two loci in chromosomes 2 and 15 that could be associated with apple fruit russet (Falginella et al., 2015; Lashbrooke et al., 2015). QTL analysis in kiwifruit identified two types of gene loci that either associated with russet formation or were involved in cuticle integrity and coverage (Macnee et al., 2021). These results indicate the complexity of russet regulation and the presence of different determinants for the trait formation within and among different species.

## Molecular triggers in fruit periderm development

Periderm development can be either repressed by a strong cuticle protection or advanced by a strong elicitor (**Figure 1**). Construction of cuticle membrane by multiple biopolymers suggests a complex regulation network underlying the development of the tissue. Previous studies have mapped different loci controlling cuticle strength or the propensity to russet in fruit skin (Lashbrooke et al., 2015; Macnee et al., 2021; Jiang et al., 2022). Orthologues of WAX INDUCER1/SHINE1 (SHN1/WIN1) have been shown to regulate cuticle formation in different species (Aharoni et al., 2004; Broun et al., 2004; Kannangara et al., 2007; Shi et al., 2011; Shi et al., 2013; Hen-Avivi et al., 2014). In apple, MdSHN3 was revealed to have an extremely low expression, which is in line with cuticle failure and russet formation, and could act as a putative negative regulator in apple fruit periderm development (Lashbrooke et al., 2015). *PyPPCD1* has been identified as a strong elicitor dominantly controlling the periderm development in pear fruit skin by triggering an ARF1-dependent PCD and dedifferentiation of the parenchymal cells into cork cambium (Wang et al., 2022). *PyPPCD1* has shown a preferential expression in pear fruit skin during the periderm development and its orthologues have been proved to be conservative with PCD function in different species (Wang et al., 2022). The environmental stress-inducible genes and quantitative traits suggest that there are more molecular regulators in the periderm development. Several key genes encoding the catalytic enzymes acyltransferases and P450s, and the transcription factor MYB1 and NAC for either suberin or cutin biosynthesis have been identified in other organs and species (Li et al., 2007; Höfer et al., 2008; Serra et al., 2009; Capote et al., 2018; Soler et al., 2020). These genes showed effect on the composition and structure of the periderm or cuticle. Further studies are still needed to determine whether these genes have a switch role in periderm development.

## The common regulation in periderm

**Figure 3:**
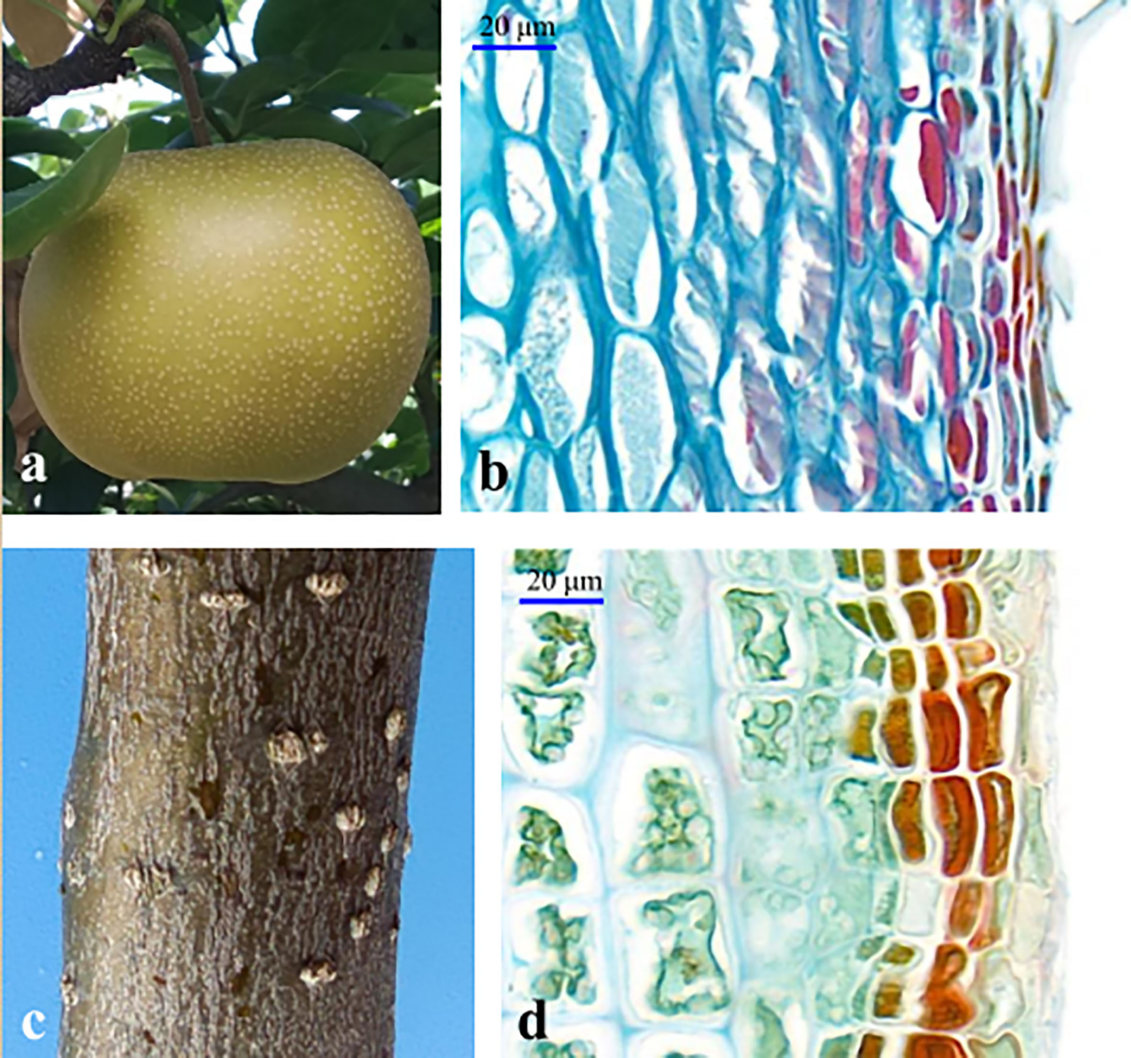
The periderm development in sand pear fruit **(A, B)** and stem **(C, D)**.

Suberized cells form the periderm. Suberins are complex polyesters which are highly variable in the relative proportion of the different families of monomers, and of the individual suberin acids depending on plant species and tissue location (reviewed by Graça, 2015; Woolfson et al., 2022). Although showing specific characters with a visible structure in the outmost cork layer (**Figure 1**), periderm shares the conserved developmental processes and conservative regulation among different species and organs (Lashbrooke et al., 2016; Fernández-Piñán et al., 2021; Serra et al., 2022). PCD is a process that causes an actively induced and tightly controlled cellular suicide, and different types of PCD play crucial roles in plant development, as well as in the reaction to environmental stresses (Daneva et al., 2016). Previous studies mainly described PCD as the result of environmental stress or as the destiny of the suberizing cells in the periderm development, while its regulatory role during the development of the tissue was not clearly indicated. Pear fruit is an annual organ, whereas its periderm development is similar to that of the perennial stem (**Figure 3**), which suggests the similar regulatory mechanisms underlying the development of the two types of the tissue. Thus, the regulatory role of PCD in pear fruit periderm development is supposed to be conservative in the development of other types of periderm.

**Figure 4:**
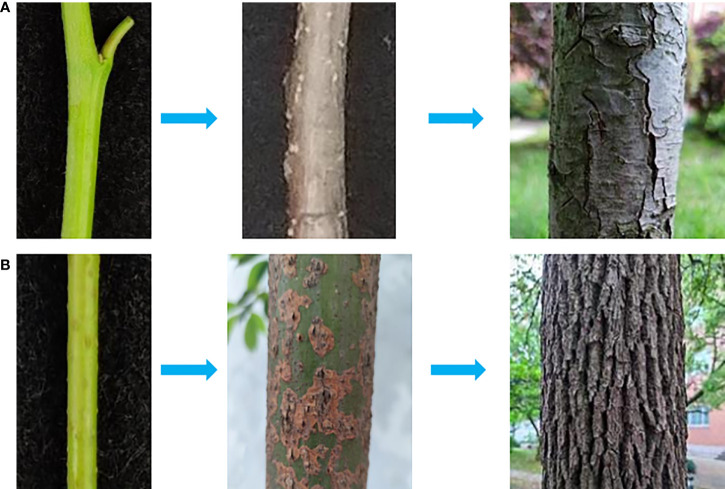
Different types of stem periderm. In the direction of the arrow, the new spring stem, biennial stem, and perennial stem of sand pear (*Pyrus* pyrifolia) **(A)** and camphor tree (*Camphora officinarum*) **(B)** could be observed.

**Figure 5:**
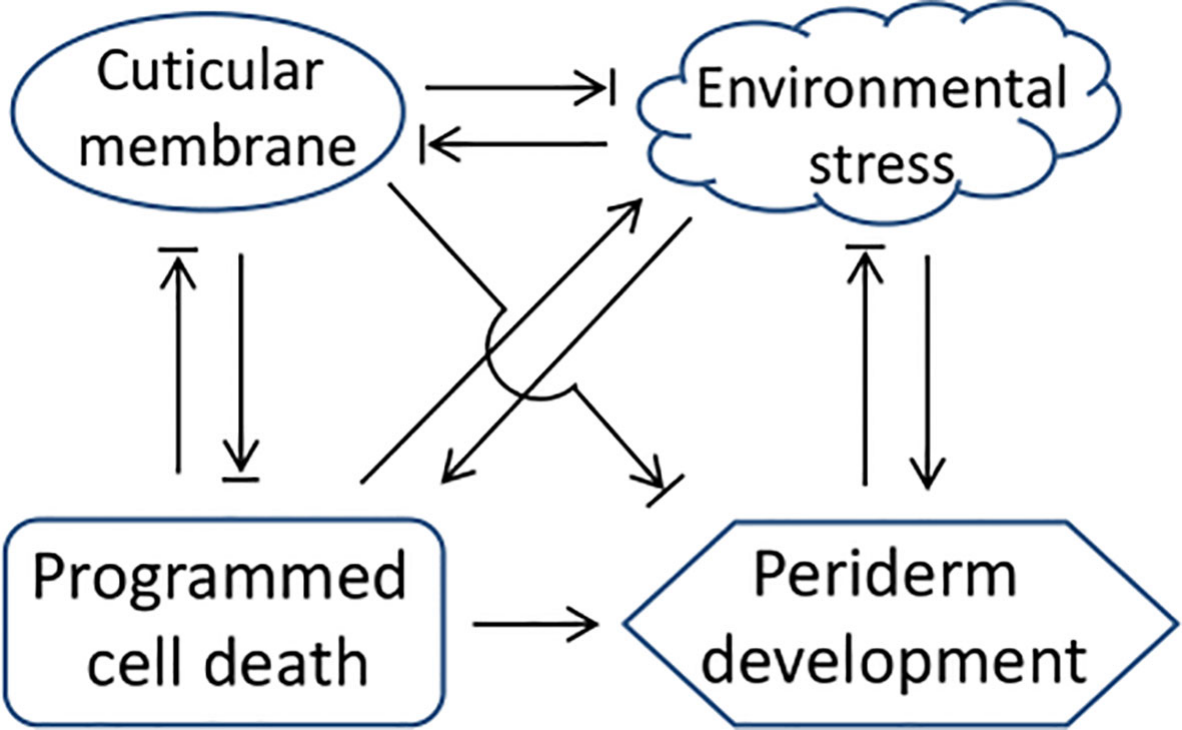
A network of the regulatory factors and periderm development. The arrow and termination arrow symbols indicate that one side of the link promotes or inhibits the development of the other side.

In addition to the PCD-triggered type, uneven development of periderm also exists in some tree stems (**Figure 4**), which is similar to the semi-russet type periderm of pear fruit. For tree stem and other perennial organs, their periderm meristem typically shows perennial properties with seasonal rhythm and stress responsiveness, and the timing of the first periderm formation also varies among different species from the first year of growth to several years delayed (Serra et al., 2022). The cuticle serves as a primary protective layer to protect plant organs from environmental stresses that induce periderm development, while the genetic persistence of cuticle membrane is different among different species (Lashbrooke et al., 2015; Macnee et al., 2021), which leads to the protection variation and causes differences in the occurrence of the first periderm among different species. In order to better understand the regulation of periderm development, we summarized and plotted the regulatory relationship between periderm development and the regulatory factors based on the above-mentioned analysis (**Figure 5**), which is generally considered for the regulation of periderm development in different organs or species. The molecular triggers identified most recently in pear and apple showed functional conservation among their homologs (Lashbrooke et al., 2015; Wang et al., 2022), their certain roles for periderm development in different types of organs and species are still needed to be clarified in the next studies.

## Conclusions

The present review summarized a putative common pathway for periderm development, i.e. either environmental stress or PCD could disrupt the structure of plasma membrane and other organelles, leading to the cells at the condition of oxygen exposure and water imbalance, followed by the stress response with activation of the synthesis of phytohormones to initiate phellogen and periderm development. PCD has been revealed to play a key role in the regulation of periderm development (Wunderling et al., 2018; Wang et al., 2022), which is also shared by the vascular cambium in its differentiation to xylem (Wunderling et al., 2018; Serra et al., 2022). The β-glucuronidase (GUS) fusion expression analysis indicated that the *PyPPCD1* gene was also highly expressed in the vascular cambium of a poplar system (data were not published). Considering the role of PyPPCD1*-*guided PCD in cork cambium development regulation, this result suggests the similar regulation for the vascular cambium activity as that in cork cambium. The roles of *PyPPCD1* gene and its homologs in the co-regulation of the development of these tissues need to be further explored. The regulation of periderm development consists of a complex regulatory network superimposed by multiple aspects of regulation, including the tissue or cell type specificity, seasonal and developmental stage specificity, and environmental stress response. More regulators of phellogen proliferation and differentiation are required to be functionally characterized to refine the framework of the molecular mechanisms. Robust tools and methods, including single-cell transcriptomics and genetic manipulation, will facilitate this process.

## Author contributions

Y-ZW prepared the figures and wrote the manuscript. Z-BS, M-SD, and D-YC contributed their ideas and suggestions for the manuscript revision. All authors have read and agreed to the published version of the manuscript.

## Funding

This work was partially funded by the Earmarked Fund for China Agriculture Research System (CARS) and the Key Project for New Agricultural Cultivar Breeding in Zhe-jiang Province, China (2021C02066-5).

## Conflict of interest

The authors declare that the research was conducted in the absence of any commercial or financial relationships that could be construed as a potential conflict of interest.

## Publisher’s note

All claims expressed in this article are solely those of the authors and do not necessarily represent those of their affiliated organizations, or those of the publisher, the editors and the reviewers. Any product that may be evaluated in this article, or claim that may be made by its manufacturer, is not guaranteed or endorsed by the publisher.
